# Victor Hugo’s Presage of Immortality

**Published:** 1891-09

**Authors:** 


### VICTOR HUGO'S PRESAGE OF IMMORTALITY.

(*From L' Univers.*)

At a dinner given to Victor Hugo, in Paris, some years ago, he delivered an impromptu address, in which he gave expression to his faith in the Infinite and in the soul's immortality.

We think, perhaps, we have published this before, but if so, it will bear repeating, yea, and many times over.

This is what the distinguished French poet and philosopher said :

"I am rising toward the sky. The sunshine in on my head. Winter is on my head and eternal Spring is in my heart. There I breathe at this hour the fragrance of the lilacs, the violets and roses as at twenty years ago. The nearer I approach the end the plainer I hear around me the immortal symphonies of the worlds which invite me.

"It is marvelous, yet simple. It is a fairy tale and it is historic. For half a century I have been writing my thoughts in prose and verse, history, philosophy, drama, romance, tradition, satire, ode and song. I have tried all, but I feel I have not said a thousandth part of what is in me. When I go down to the grave I can say, like many others, I have finished my day's work; but I can not say I have finished my life. My days will begin again the next morning. The tomb is not a blind alley; it is a thoroughfare. It closes on the twilight to open on the dawn."

